# Bronchoscopy-Guided Intratracheal Suture Repair of Recurrent Tracheoesophageal Fistula in an Adolescent

**DOI:** 10.3390/jcm15156126

**Published:** 2026-08-06

**Authors:** Lu Qin, Xiao-Fen Tao, Jia Liu, Ting Huang, Hu-Jun Wu, Guo-Ping Jin, Lan-Fang Tang, Lei Wu

**Affiliations:** 1 Department of Pulmonology, Children’s Hospital, Zhejiang University School of Medicine, National Clinical Research Center for Children and Adolescents’ Health and Diseases, Hangzhou 310052, China; 21918496@zju.edu.cn (L.Q.); icyshiny@zju.edu.cn (X.-F.T.);; 2 Department of Otolaryngology, Children’s Hospital, Zhejiang University School of Medicine, Hangzhou 310052, China; 6512057@zju.edu.cn; 3 Department of Thoracic Surgery, Children’s Hospital, Zhejiang University School of Medicine, Hangzhou 310052, China; 6512045@zju.edu.cn; 4 Department of Endoscopy Center, Children’s Hospital, Zhejiang University School of Medicine, Hangzhou 310052, China

**Keywords:** tracheoesophageal fistula, esophageal atresia, interventional pulmonology, endoscopic suturing, bronchoscopy

## Abstract

**Introduction:** Managing recurrent congenital tracheoesophageal fistula (rTEF) is clinically challenging. Repeat thoracotomy is technically demanding due to dense adhesions and carries high morbidity, whereas outcomes of conventional endoscopic interventions vary, and sustained closure may be difficult to achieve in complex recurrent fistulas. We report the use of bronchoscopy-guided intratracheal suturing in a refractory case. **Case Presentation:** A 17-year-old male with a history of congenital TEF experienced multiple recurrences despite two thoracotomies and one endoscopic clipping attempt. The procedure was performed under general anesthesia without endotracheal intubation, and superimposed high-frequency jet ventilation was used for airway management. Following electrocautery treatment of the visible tracheal-side margins, four interrupted absorbable sutures were placed through tissues adjacent to the fistula opening under bronchoscopic guidance. Tightening of the sutures approximated the visible fistula margins. The procedure lasted 62 min with no complications. Follow-up bronchoscopy, methylene blue testing, and esophagography at 2 weeks showed no evidence of fistula leakage, indicating early technical closure. At the 3-month telephone follow-up, the patient remained asymptomatic and reported normal oral intake and physical activity. These findings suggest early technical success; longer objective follow-up is required to assess durability. **Conclusions:** In this highly selected adolescent, bronchoscopy-guided intratracheal suturing was technically feasible and achieved early fistula closure without a perioperative complication. Longer objective follow-up after loss of suture tensile strength is required to determine whether closure can be maintained without mechanical suture support.

## 1. Introduction

Congenital esophageal atresia with tracheoesophageal fistula (EA-TEF) is a significant congenital anomaly, with an incidence ranging from 1 in 3000 to 1 in 3500 live births [1]. Type C is the most common variant [2]. Owing to advancements in pediatric surgery, cure rates now exceed 90%. As a result, the clinical focus has shifted toward managing postoperative complications and improving long-term quality of life [3]. Among these complications, recurrent tracheoesophageal fistula (rTEF) remains one of the most serious challenges, with a reported incidence of 3% to 14% after primary repair [4,5]. Recurrent fistulas rarely heal spontaneously and often cause persistent aspiration and pulmonary infections.

Repeat thoracotomy carries substantial risks because of severe adhesions and distorted anatomy. Endoscopic alternatives include covered self-expanding metal stents, over-the-scope clips, and various tissue sealants. However, stenting is largely palliative, and frequent maintenance bronchoscopies pose a considerable burden on fragile patients. There is therefore a need to explore less invasive approaches for selected patients in whom repeat thoracotomy carries substantial risk. Endoscopic suturing permits direct mechanical tissue apposition while avoiding surgical re-entry into a previously operated thoracic field. Herein, we describe a bronchoscopy-guided intratracheal suture repair in an adolescent with multiply recurrent TEFs who failed two prior surgical revisions and one endoscopic clipping attempt. This report describes the procedural technique and illustrates its feasibility and early technical outcome in a highly selected adolescent with multiply recurrent TEF after failed surgical and endoscopic interventions.

## 2. Case Presentation

**Figure 1:**
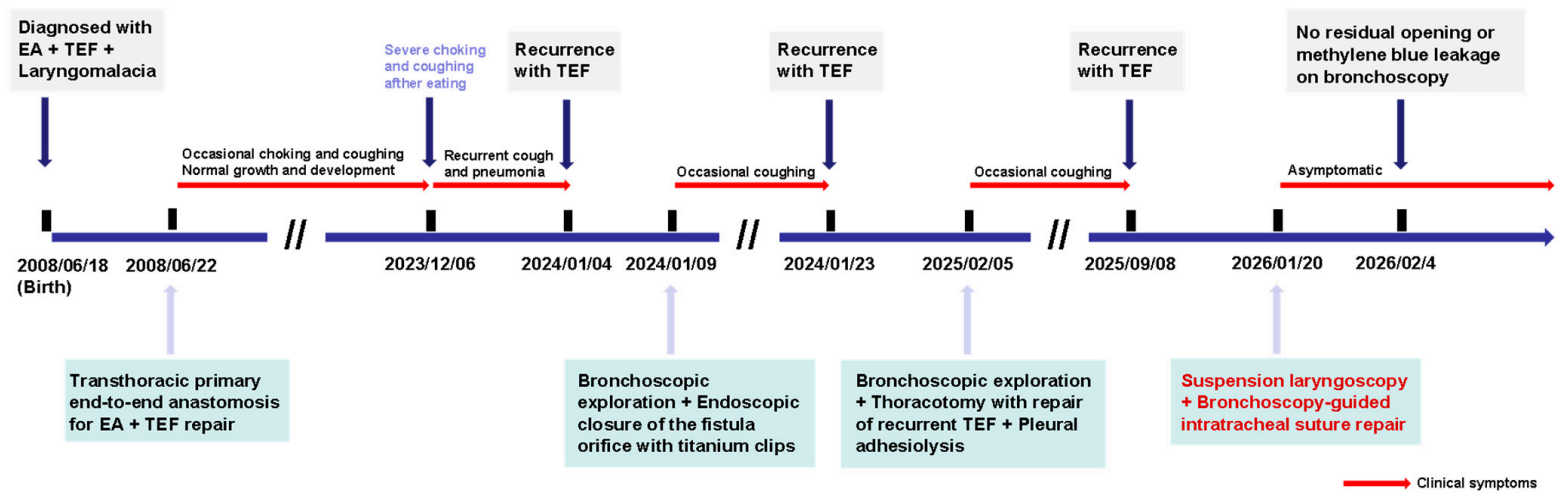
Clinical timeline of the recurrent tracheoesophageal fistula and its management. Recurrent TEF was confirmed on 4 January 2024, and endoscopic clip closure was performed on 9 January 2024. Recurrence was documented on 23 January 2024. Repeat surgical repair was performed on 5 February 2025, but persistent rTEF was demonstrated on 8 September 2025. Bronchoscopy-guided intratracheal suturing was performed on 20 January 2026. Follow-up bronchoscopy and methylene blue testing performed approximately 2 weeks after bronchoscopy-guided intratracheal suturing showed no visible residual opening or dye leakage, supporting early technical closure. Long-term anatomical healing was not established. These findings represent early technical closure rather than confirmed long-term healing.

**Figure 2:**
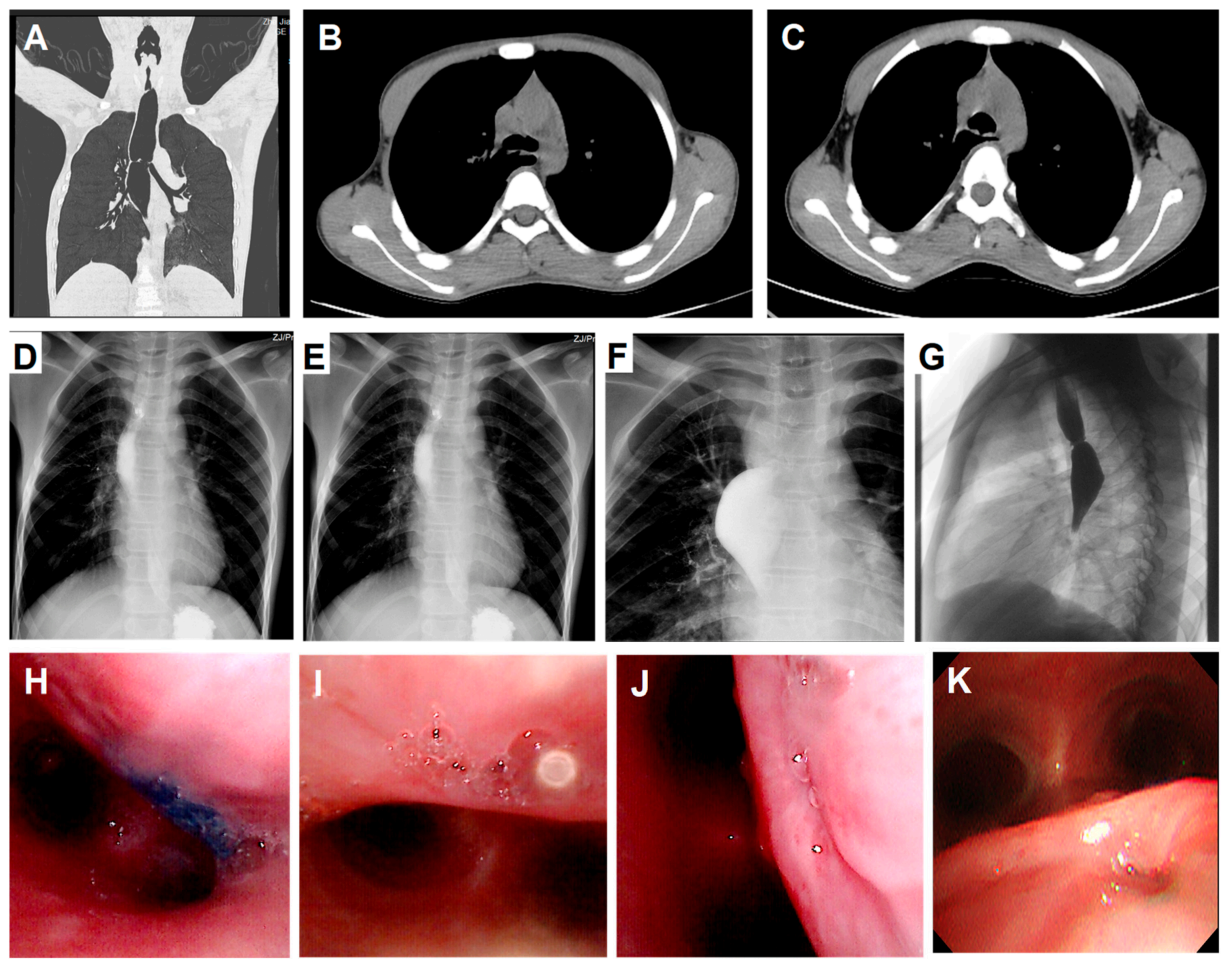
Serial radiological and bronchoscopic findings of the recurrent tracheoesophageal fistula (rTEF) across the treatment course. (**A**) Initial three-dimensional coronal CT reconstruction in January 2024 demonstrating the fistulous tract between the anterior esophageal wall and the posterior trachea. (**B**) Axial CT in March 2024 confirming a persistent fistulous tract after the failed endoscopic clipping. (**C**) Axial CT in September 2025 showing the persistent rTEF after the second open thoracotomy. (**D**,**E**) Serial esophagograms after esophageal-side clip closure showing retained titanium clips adjacent to the esophageal opening, localized esophageal narrowing, and persistent contrast leakage through the fistula into the right bronchial tree. (**F**) Esophagogram after the second thoracotomy demonstrating massive contrast extravasation into the airways, indicating surgical failure. (**G**) Lateral esophagogram obtained 2 weeks after bronchoscopy-guided intratracheal suture repair, showing no contrast leakage. (**H**) Initial bronchoscopy showing methylene blue dye leakage from the fistula orifice on the posterior membranous wall after instillation through a nasogastric tube. (**I**) Bronchoscopy after esophageal-side clip closure showing persistent bubbling secretions from the tracheal opening above the carina, indicating continued fistula patency. (**J**,**K**) Serial bronchoscopies before and after the second thoracotomy still demonstrating the persistent fistula with continuous leakage of white foamy liquid.

A 17-year-old male with congenital Gross type C esophageal atresia with tracheoesophageal fistula (EA-TEF) and laryngomalacia underwent primary transthoracic repair during the neonatal period, 4 days after birth (22 June 2008). He remained clinically stable for more than 15 years, with only occasional coughing and normal growth and development, although no regular follow-up was conducted (Figure 1). On 6 December 2023, he developed severe choking and coughing after eating, followed by three episodes of recurrent pneumonia. Approximately 1 month later, on 4 January 2024, chest computed tomography (CT) (Figure 2A) and bronchoscopy (Figure 2H) confirmed rTEF. Initial endoscopic closure was performed from the esophageal side 5 days later, on 9 January 2024, using four titanium clips to approximate the esophageal opening of the fistula.

Clinical recurrence was noted 14 days after clip placement, on 23 January 2024. Subsequent esophagography, CT, and bronchoscopy performed in March 2024, approximately 2 months after the clipping procedure, confirmed persistent fistula patency, with retained esophageal-side clips, progressive contrast leakage into the right bronchial tree, a persistent fistulous tract, and bubbling secretions from the tracheal opening (Figure 2B,D,E,I). Approximately 13 months after the endoscopic clipping procedure, the patient underwent repeat thoracotomy with surgical repair on 5 February 2025. Nevertheless, repeat evaluation approximately 7 months later, on 8 September 2025, continued to demonstrate persistent rTEF (Figure 1; Figure 2C,F,J,K). Given the failure of the previous surgical and endoscopic interventions, bronchoscopy-guided intratracheal suturing was performed approximately 4 months later, on 20 January 2026.

### 2.1. Bronchoscopic Suture Repair Procedure

The operating team was composed of three therapeutic endoscopists, one thoracic surgeon, one anesthesiologist, and two circulating nurses. The procedure was undertaken as individualized clinical treatment following multidisciplinary review and was not performed as part of a prospectively initiated research protocol.

The procedure was performed under general anesthesia without endotracheal intubation. A nasogastric tube was placed before the procedure for gastric decompression. Airway management was maintained using superimposed high-frequency jet ventilation (TwinStream, CARLREINER, Vienna, Austria). The normal-frequency component was set at 12 cycles/min with an inspiratory-to-expiratory ratio of 1:1 and a driving pressure of 0.5 bar. The high-frequency component was set at 600 cycles/min with an inspiratory-to-expiratory ratio of 1:1.5 and a driving pressure of 0.4 bar. The FiO_2_ setting was not retained in the available anesthesia record. Oxygenation was continuously monitored, and the chest and abdomen were repeatedly assessed for abnormal expansion or distension that might indicate gas passage through the fistula. No abnormal chest expansion or abdominal distension was observed during the procedure. A self-retaining laryngoscope (CTNS-360-K41, Tiansong Medical, Hangzhou, China) was then inserted. The self-retaining laryngoscope established a stable operating field and was used to reduce repeated direct contact between the rigid surgical instruments and the vocal cords. Afterward, a videobronchoscope (Model B4020, Zhuhai Joyrun Medical Technology Co., Ltd., Zhuhai, China) was advanced into the trachea to clearly visualize the fistula. A discrete tracheal opening was identified on the posterior membranous wall approximately 13 cm from the incisors and 2 cm above the carina. The opening led to a patent fistulous tract that communicated directly with the anterior esophagus rather than terminating as a blind sinus. The diameters of the tracheal and esophageal openings and the length of the fistulous tract were not formally measured during the procedure. The operative video contained no calibrated scale that would permit reliable retrospective measurement. No tissue biopsy was obtained; therefore, the extent of tract epithelialization was not histologically assessed. Electrocautery was applied from the tracheal side to the visible margins of the tracheal opening to create a fresh tissue surface. This treatment was intended to disrupt the epithelial lining at the visible tracheal margins. Complete de-epithelialization was not histologically verified, and neither the entire fistulous tract nor the esophageal-side margins were ablated.

**Figure 3:**
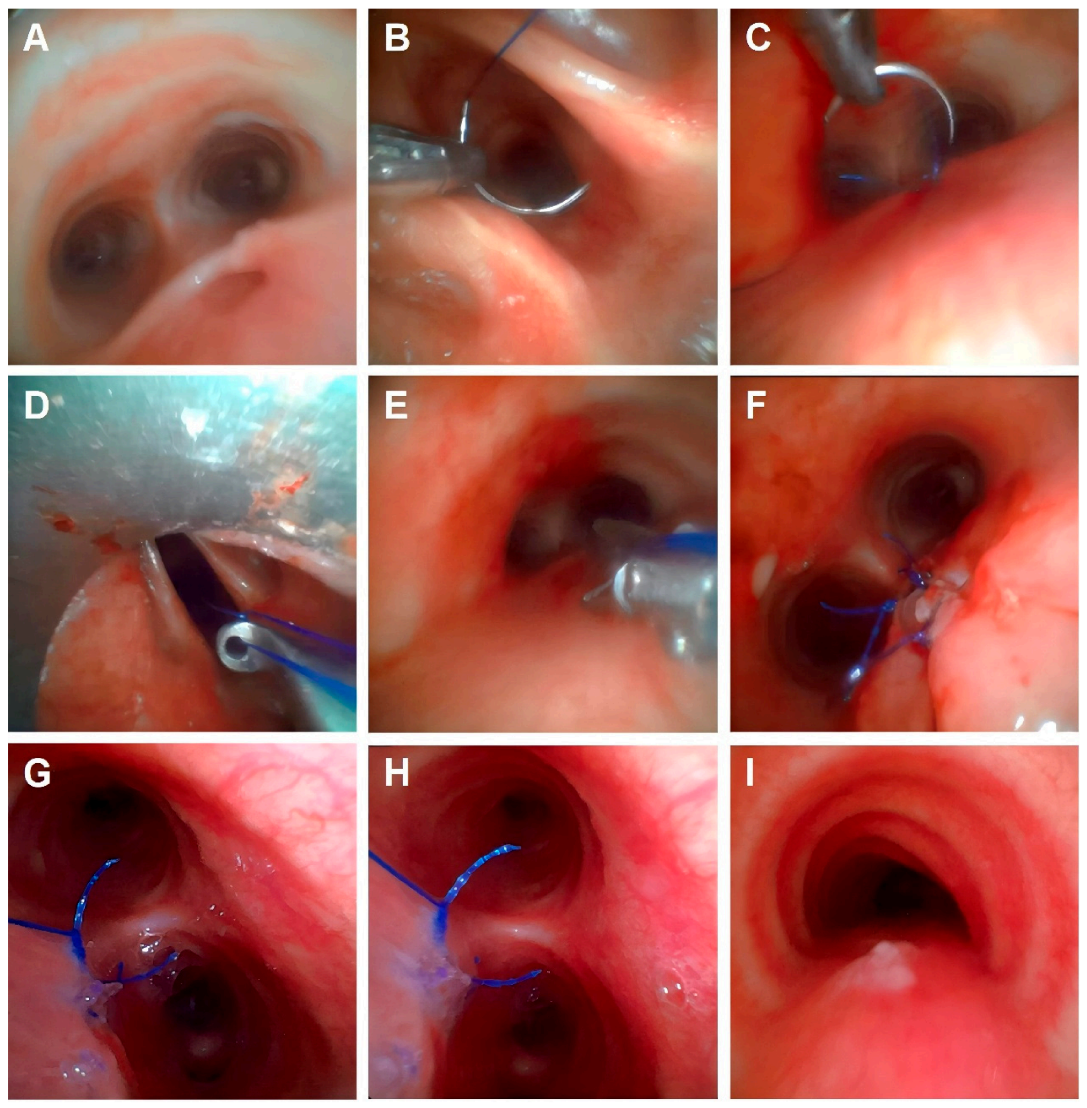
Bronchoscopic suture procedure and 2-week follow-up bronchoscopy. (**A**–**F**) A fistula orifice located approximately 13 cm from the incisors and 2 cm above the carina was identified on the posterior membranous wall. Under direct bronchoscopic guidance, a needle holder was introduced into the tracheal lumen to place four interrupted 4–0 and 5–0 absorbable sutures. The needle entry and exit points were visualized from the tracheal lumen. Tightening of the four interrupted sutures approximated the visible margins of the tracheal opening. The exact depth of the bites could not be determined from the video. (**G**–**I**) Follow-up bronchoscopy at 2 weeks showed that the tracheal opening remained apposed, with no residual opening visible; the absorbable sutures remained in situ. After methylene blue was instilled through a nasogastric tube, no leakage into the tracheal lumen was observed.

Endoluminal suture closure was then performed under direct bronchoscopic guidance: a rigid thoracoscopic needle holder, grasping the suture needle, was introduced into the trachea alongside the bronchoscope. A total of four interrupted sutures were placed through the tissues adjacent to the fistula opening using 4–0 and 5–0 absorbable materials (Biosyn™, Covidien, Mansfield, MA, USA). The needle entry and exit points were directly visualized from the tracheal lumen. However, the exact depth of the bites and the individual tissue layers incorporated could not be determined from the operative video and were not specified in the contemporaneous operative record. Owing to the restricted working space, the knots were tied sequentially outside the oral cavity and advanced to the fistula site using a rigid knot pusher. Tightening of the sutures approximated the visible margins of the tracheal opening, after which the suture tails were trimmed with endoscopic scissors (Figure 3A–F). Upon completion of the suturing, secretions were aspirated from the airway. After completion of the tracheal suturing, the same videobronchoscope was withdrawn to the pharynx and advanced transorally into the esophagus. During gentle air insufflation, the esophageal-side opening appeared apposed, and no obvious bubbling was observed. This was considered an immediate qualitative assessment rather than evidence of durable closure. Finally, the bronchoscope was withdrawn. The total procedure time was 62 min. No intraprocedural complications were observed.

### 2.2. Postoperative Management and Follow-Up

Postoperative feeding was individualized because no standardized institutional protocol was available for this procedure. Oral intake was withheld for the first 24 h, during which parenteral nutritional support was provided. The 24 h period was selected by the treating team as an individualized observation interval rather than on the basis of a validated feeding protocol. As the patient remained clinically stable without symptoms suggestive of leakage, a liquid diet was started after 24 h. This timing should not be interpreted as a standardized recommendation for other patients. On postoperative day 13, esophagography showed no contrast leakage into the airway (Figure 2G). The examination also demonstrated occasional small-volume reflux of contrast into the esophagus, which was interpreted as mild gastroesophageal reflux. At approximately 2 weeks, videobronchoscopy showed that the tracheal opening remained apposed, with no residual opening visible (Figure 3G–I). Bilateral vocal cord mobility was normal, and the patient had no hoarseness, stridor, or swallowing difficulty. Methylene blue was then instilled into the esophagus through a nasogastric tube, with no dye observed in the tracheal lumen. The patient subsequently resumed a normal diet without feeding-related adverse events. At the 3-month telephone follow-up, he remained asymptomatic and reported no limitation in daily diet or physical activity.

## 3. Discussion

This report describes the technical feasibility and early outcome of bronchoscopy-guided intratracheal suturing in a highly selected adolescent with TEF after multiple failed interventions. It does not establish comparative efficacy or support routine use in broader patient populations. Because no anatomical surveillance was performed after the neonatal repair, the timing of fistula formation remains uncertain. The late presentation may represent delayed recurrence or a previously occult residual fistula that later became symptomatic.

In this patient, two previous thoracotomies resulted in dense adhesions and distorted anatomy, which increased the risk of another open repair. The intratracheal approach provided direct access to the fistula without re-entering the previously operated thoracic field. Other endoscopic treatments include covered self-expanding metal stents, over-the-scope clips, tissue adhesives, and thermal techniques [6,7,8,9,10]. Stents and clips may cause bleeding, migration, granulation tissue formation, or poor tolerance [11,12]. Intratracheal suturing also has specific risks, including suture-related granulation, secretion retention, and focal airway stenosis, and therefore requires long-term surveillance.

In our patient, the previous endoscopic procedure used titanium clips to approximate only the esophageal opening but did not include electrocautery treatment of the fistula margins or suture-mediated tissue apposition, and sustained closure was not achieved. The present procedure combined electrocautery treatment of the visible tracheal-side margins with interrupted sutures placed through adjacent tissues. This method was intended to create a fresh tissue surface and maintain mechanical apposition during early healing. We hypothesized that local epithelial disruption and sustained tissue contact might promote granulation and fibrosis. However, complete de-epithelialization was not histologically confirmed, and the exact depth and tissue layers included in the suture bites could not be determined from the operative documentation. The entire fistulous tract and the esophageal-side margins were not ablated. The proposed healing mechanism therefore remains hypothetical, and permanent anatomical obliteration cannot be inferred from the available follow-up.

In this case, a self-retaining laryngoscope was used to maintain glottic exposure, while a flexible videobronchoscope provided visualization and rigid suturing instruments were introduced alongside it. This arrangement was selected to preserve intratracheal working space for independent manipulation of the videobronchoscope and needle holder and to permit directed visualization of the posterior fistula near the carina. The choice was specific to this patient’s airway caliber, fistula location, and instrument configuration and should not be interpreted as evidence that this approach is preferable to rigid bronchoscopy. Rigid bronchoscopy is also a viable access platform for endobronchial TEF suturing, as illustrated by the Galluccio series discussed below. Selection of the access platform should be individualized according to airway anatomy, fistula location, instrument dimensions, ventilation requirements, and operator experience. In the presence of an open TEF, however, jet ventilation may divert gas into the esophagus and stomach, with risks of gastric distension, barotrauma, and inadequate ventilation or oxygenation. Gastric decompression, continuous oxygenation monitoring, and observation of chest and abdominal expansion are therefore necessary. The four interrupted absorbable sutures provided temporary mechanical apposition of the visible fistula margins. Tensile strength loss and material absorption are distinct processes. Published data indicate that Biosyn loses approximately half of its tensile strength within 14 to 21 days and all tensile strength by approximately 28 days, whereas complete mass absorption occurs over approximately 90 to 110 days [13]. The objective assessments at approximately 2 weeks were therefore performed while the sutures still provided mechanical support. The 3-month follow-up was telephone-based and did not include bronchoscopy or imaging. Anatomical closure after loss of suture tensile strength was therefore not verified, irrespective of whether residual suture material remained present.

The term “transtracheal” has previously been used to describe a different surgical approach. Ambrogi et al. [14] reported open repair of a large membranous laceration of the thoracic trachea rather than a tracheoesophageal fistula. Their procedure involved a low cervical collar incision followed by a longitudinal anterior tracheotomy, through which the membranous tear was exposed and repaired with a running suture. The tracheotomy was then closed with interrupted crossed sutures. Although this approach avoided thoracotomy, it remained an open transcervical operation and should not be regarded as a direct precedent for bronchoscopic TEF closure.

A relevant procedural precedent was reported by Galluccio et al., who performed endobronchial suturing through rigid bronchoscopy in ten patients with non-neoplastic TEFs without tracheostomy [15]. Their series supports rigid bronchoscopy as a viable access platform for airway-based TEF suturing. Four patients achieved sustained closure after a single procedure. Five patients underwent repeat endoscopic suturing after partial reopening, and 17 procedures were performed in total. At 1 year, complete closure was reported in nine patients, whereas one patient with complete reopening was referred for surgical treatment. These findings support technical feasibility but also indicate that repeat intervention was frequently required. Their cohort consisted predominantly of adults with acquired fistulas treated using rigid bronchoscopy. In the present case, the patient was an adolescent with a congenital postoperative TEF, and rigid suturing instruments were introduced alongside a flexible videobronchoscope that provided visualization and guidance. Differences in etiology, age, tissue characteristics, fistula anatomy, and instrumentation limit direct comparison. The results of the Galluccio series therefore cannot establish the efficacy or durability of this procedure in congenital recurrent TEF. The present report represents an adaptation of a previously described airway-based suturing concept to a different clinical and technical setting rather than a wholly novel treatment principle. Several features made intratracheal suturing technically possible in this patient. His airway was sufficiently wide to accommodate the bronchoscope and rigid suturing instruments simultaneously. The fistula presented as a discrete tracheal opening on the posterior membranous wall approximately 2 cm above the carina and communicated directly with the esophagus through a patent tract. Its position allowed direct visualization, electrocautery treatment of the visible tracheal margins, bronchoscopically guided needle placement, and approximation of the fistula margins. However, the opening diameters and tract length were not formally measured, which limits anatomical characterization and reproducibility. Previous endoscopic and surgical treatments had failed, and another open repair was considered particularly difficult because of the previously operated thoracic field. The procedure was also supported by a multidisciplinary team experienced in therapeutic bronchoscopy, thoracic surgery, and high-frequency jet ventilation. These characteristics may assist case selection but should not be regarded as established indications.

We also acknowledge the inherent limitations of this report. A single case cannot establish efficacy, safety, or generalizability, and the available follow-up does not confirm anatomical closure after loss of mechanical suture support. The diameters of the fistula openings and the tract length were not formally measured, and the absence of a calibrated scale in the operative video precluded reliable retrospective measurement. Technical feasibility depends on airway caliber, fistula size and location, tissue quality, and the ability to obtain a stable approach. A narrow airway, a large or poorly defined fistula, an unfavorable angle, or inflamed, fibrotic, friable, or insufficient tissue may prevent safe needle placement and tension-free apposition. Proximity to critical airway structures, uncontrolled bleeding, interference with ventilation, or inadequate oxygenation may require the procedure to be discontinued or converted to another treatment. Intraluminal knots and local tissue injury may subsequently cause granulation, secretion retention, scarring, posterior membranous wall deformity, or focal tracheal stenosis. Long-term bronchoscopic surveillance is therefore required, with chest imaging or esophagography when clinically indicated. If closure fails or the fistula recurs, management should be reconsidered by a multidisciplinary team according to the anatomy, tissue quality, previous interventions, and clinical condition. Possible options include repeat endoscopic treatment, selected clipping or occlusion, stenting as a bridge or palliative measure, and repeat surgical repair. The procedure also required three therapeutic endoscopists, a thoracic surgeon, an anesthesiologist experienced in high-frequency jet ventilation, two nurses, and specialized rigid instruments. These requirements may restrict its use to experienced tertiary referral centers, and its feasibility with fewer personnel or less specialized equipment remains unknown.

## 4. Conclusions

Bronchoscopy-guided intratracheal suturing was technically feasible and achieved early closure in this highly selected adolescent with recurrent TEF after multiple failed interventions. Because objective evaluation was limited to approximately 2 weeks and no anatomical assessment was performed after the expected loss of suture tensile strength, the durability and safety of this technique cannot be determined from this case.

## Data Availability

The data that support the findings of this study are not publicly available owing to privacy and ethical restrictions but are available from the corresponding author upon reasonable request.

## References

[1] Cassina M., Ruol M., Pertile R., Midrio P., Piffer S., Vicenzi V., Saugo M., Stocco C.F., Gamba P., Clementi M. (2016). Prevalence, characteristics, and survival of children with esophageal atresia: A 32-year population-based study including 1,417,724 consecutive newborns. Birth Defects Res. Part A Clin. Mol. Teratol..

[2] van Lennep M., Singendonk M.M.J., Dall’Oglio L., Gottrand F., Krishnan U., Terheggen-Lagro S.W.J., Omari T.I., Benninga M.A., Van Wijk M.P. (2019). Oesophageal atresia. Nat. Rev. Dis. Prim..

[3] Holland A.J., Fitzgerald D.A. (2010). Oesophageal atresia and tracheo-oesophageal fistula: Current management strategies and complications. Paediatr. Respir. Rev..

[4] Wang J., Zhang M., Pan W., Wu W., Yan W., Cai W. (2017). Management of recurrent tracheoesophageal fistula after esophageal atresia and follow-up. Dis. Esophagus.

[5] Thakkar H.S., Hewitt R., Cross K., Hannon E., De Bie F., Blackburn S., Eaton S., McLaren C.A., Roebuck D.J., Elliott M.J. (2019). The multi-disciplinary management of complex congenital and acquired tracheo-oesophageal fistulae. Pediatr. Surg. Int..

[6] Schlemmer C., Voigtländer T., Drews J., Engelke C., Marquardt J.U., Heidrich B., Klein F., Wedemeyer H., Kirstein M.M., von Hahn T. (2024). Safety and efficacy of conventional compared to segmented esophageal fully covered self-expanding metal stents: A retrospective multicenter case-control study. Surg. Endosc..

[7] Vinnamala S., Murthy B., Parmar J., Bhasin N., Verma P., Melville C., Mannath J. (2014). Rendezvous technique using bronchoscopy and gastroscopy to close a tracheoesophageal fistula by placement of an over-the-scope clip. Endoscopy.

[8] Ramai D., Bivona A., Latson W., Ofosu A., Ofori E., Reddy M., Adler D.G. (2019). Endoscopic management of tracheoesophageal fistulas. Ann. Gastroenterol..

[9] Richter G.T., Ryckman F., Brown R.L., Rutter M.J. (2008). Endoscopic management of recurrent tracheoesophageal fistula. J. Pediatr. Surg..

[10] Gregory S., Chun R.H., Parakininkas D., Amos L., Fons R., Lerner D.G., Lal D.R., Sulman C. (2017). Endoscopic esophageal and tracheal cauterization for closure of recurrent tracheoesophageal fistula: A case report and review of the literature. Int. J. Pediatr. Otorhinolaryngol..

[11] Jeong B.H., Ng J., Jeong S.H., Kim H. (2020). Clinical outcomes of complications following Self-Expandable Metallic Stent Insertion for benign tracheobronchial stenosis. Medicina.

[12] Zakaluzny S.A., Lane J.D., Mair E.A. (2003). Complications of tracheobronchial airway stents. Otolaryngol. Head. Neck Surg..

[13] Greenberg J.A., Clark R.M. (2009). Advances in suture material for obstetric and gynecologic surgery. Rev. Obstet. Gynecol..

[14] Ambrogi M.C., Mussi A., Ribechini A., Angeletti C.A. (2001). Posterior wall laceration of the thoracic trachea: The transcervical-transtracheal approach. Eur. J. Cardiothorac. Surg..

[15] Galluccio G., D’Agnano V., Menichini I., Napolitano A.G., Masi U., Bianco A. (2025). Endobronchial suture of tracheoesophageal fistula through rigid bronchoscopy without tracheostomy: A preliminary, observational retrospective study. J. Clin. Med..

